# Preterm prelabour rupture of membranes (PPROM) and pregnancy outcomes in association with HIV-1 infection in KwaZulu-Natal, South Africa

**DOI:** 10.1186/s12884-020-02911-1

**Published:** 2020-04-09

**Authors:** Chidebere E. Onwughara, Dhayendre Moodley, Nthabiseng Valashiya, Motshedisi Sebitloane

**Affiliations:** Department of Obstetrics and Gynaecology, School of Clinical Medicine, University of KwaZulu-Natal, Durban, South Africa

**Keywords:** HIV-1, Preterm birth, Prelabour rupture of membranes, Pregnancy outcomes

## Abstract

**Background:**

SubSaharan Africa has a disproportionate burden of HIV and preterm births (PTB). We hypothesized that PTB in HIV-1 infected women are more likely a result of prelabour rupture of membranes (PROM) and could lead to worse birth outcomes than HIV-uninfected women. We also hypothesized that PPROM increased the risk of mother-to-child transmission (MTCT) of HIV-1. Current clinical management protocols for PPROM do not include a differential treatment plan for HIV-infected women.

**Methods:**

The maternity register at a regional hospital in a high HIV-burden district in South Africa was reviewed to identify all preterm births over a 3 month-period in 2018. We determined the incidence of PPROM using predefined criteria. Maternal age, parity, previous pregnancy complications, antenatal care, body mass index, history of smoking or alcohol, HIV infection and syphilis were computed on chi-square contingency tables to determine risk of PPROM. Overall pregnancy outcomes that included mode of delivery, fetal survival, birth weight, gestational age and newborn apgar scores were compared between HIV-infected and HIV-uninfected women whose pregnancies were complicated by PPROM. HIV-exposed newborns are routinely tested at birth for HIV by PCR.

**Results:**

A total of 1758 deliveries were recorded for Jan-Mar, 2018, and 295 (16.8%) were preterm. Maternity charts were retrieved for 236 (80.0%) PTB; 47 of PTB (19.9%; 95%CI 15.0–25.6) were further complicated by PROM which translates to 2.7% (95%CI 1.9–3.4) of all deliveries. None of the risk variables including HIV-positive status (48.9% vs 47.6%) were different between PPROM and non-PPROM groups and the majority of women were receiving cART (94.7 and 92.0%). There were no differences in the proportion of low birth weight (RR 1.2 95%CI 0.6–2.1) or severe preterm birth (RR 1.6; 95%CI 0.9–2.9) between HIV-infected and HIV-uninfected women whose pregnancies were complicated by PPROM. None of the 22 HIV-exposed newborns in the PPROM group were HIV-infected at birth.

**Conclusion:**

The PPROM incidence is not higher among HIV-infected women and our findings suggest that HIV-infected women who are virally suppressed on cART and presenting with PPROM are less likely to transmit HIV to their infants and do not have worse birth outcomes than HIV-uninfected women.

## Background

Sub-Saharan Africa carries a disproportionate burden of HIV and preterm births before 37 completed weeks of gestation [1–3]. Preterm births in developing countries are largely classified as spontaneous preterm births due to spontaneous onset of labour or prelabour premature rupture of membranes (PPROM) [4] and PPROM is known to complicate 3% of all pregnancies and contributes to a third of all preterm births [5, 6]. Globally, preterm births are known to contribute to 50% of neonatal deaths and associated with long-term neurodevelopmental impairment among surviving neonates [2]. In South Africa, preterm labour was reported to be the main cause of nearly 23% of perinatal deaths (> 500 g) [6]. The most common risk factors for PPROM are cervicovaginal infections leading to chorioamnionitis, urinary tract infections, substance abuse including smoking, uterine distention and cervical incompetence [7–9]. Although HIV-1 infection is a well-documented risk factor for preterm births [10–13], there are not many studies that explored the association between HIV-1 infection and PPROM. In the pre-ARV era, HIV-1 in South Africa was associated with a higher rate of spontaneous preterm births [14], The aetiology of this association is not well understood, and it is also not clear whether these preterm births associated with HIV-1 were due to spontaneous preterm labour or PPROM.

Prolonged rupture of membranes in HIV-infected treatment-naïve pregnant women is a known risk for mother-to-child transmission of HIV [15, 16]. There are only 2 studies that reported on MTCT rates in women whose pregnancies were complicated by PPROM, and no MTCT was reported in women on cART with PPROM [17, 18].

Management protocols for PPROM independent of HIV-status in developing countries have remained conservative and are solely guided by the gestation at which PPROM occurs. Further evidence of the impact of HIV-1 infection on PPROM outcomes and MTCT is needed for treatment protocols to be reviewed.

We hypothesized that HIV-infected pregnant women are at an increased risk for PPROM and adverse birth outcomes when compared to HIV-uninfected women who deliver preterm. We also hypothesized that mother-to-child transmission of HIV-1 was more likely to occur in preterm births complicated by prelabour rupture of membranes (PROM) than preterm births not complicated by PROM.

## Methods

The maternity register at King Edward VIII Hospital (KEH), a tertiary hospital in Durban, KwaZulu Natal was reviewed to identify all preterm births that occurred over a 3 month-period (January 1st and March 31st, 2018). KEH provides general obstetric services to the local catchment population within a 10 km radius but also serves as a referral facility for high risk pregnancies such as previous caesarean sections, multiple pregnancies, hypertension, and diabetes seen at local primary health clinics. Women presenting with PPROM < 34 weeks to local MOUs or clinics are referred to KEH. This being an exploratory study to determine the incidence of PPROM at this facility with a delivery rate of 600 per month, antenatal HIV prevalence of 40 and 12% preterm births, we chose to investigate all preterm births over a 3-month period. The maternity charts of women who delivered before completing 37 weeks of gestation during the period were sought and obstetric history, patient demography, clinical data and pregnancy outcomes were extracted for all women who delivered between 24 and 36 weeks and 6 days of gestation. Gestational age was estimated either by the last normal menstrual date, early ultrasound before 24 weeks, or when these were not available, by late ultrasound. Pregnancy outcomes included stillbirths, early neonatal outcome and birth weight. HIV-related clinical characteristics included maternal CD4 count, antiretroviral treatment and HIV status of the newborn.

Pregnancy losses or spontaneous rupture of membrane before 24 completed weeks of gestation were excluded. PPROM was defined as spontaneous ruptured membrane at least one hour before onset of labour occurring before 37 weeks.

The study was approved by the Institutional Review Board of the University of KwaZulu-Natal.

Data were analysed using IBM SPSS Statistics for Windows, Version 26.0. (Armonk, NY). Ninety-five (95) % confidence intervals were constructed around incidence point estimates i.e. incidence of PPROM for generalizability of findings to all deliveries at this facility. We tested for normal data distribution using the Shapiro-Wilk test. With continuous variables that were not normally distributed, we report the median and IQR. Differences in frequencies of categorical demographic or clinical characteristics and birth outcomes by PPROM and HIV status were assessed using the Pearson chi-square (χ^2^) test or Fisher’s exact test if an expected cell count contains fewer than 5 observations. Odds ratio and 95% confidence intervals were calculated for categorical variables. An alpha value of 0.05 was considered significant.

## Results

A total of 1758 deliveries were recorded between January 1st and March 31st, 2018, and 295 (16.8%) were preterm. For this analysis, only the preterm deliveries were included. Complete maternity charts were retrieved for 236 (80.0%) women who delivered < 37 weeks gestation. Using the limited data from the maternity register (gestational age at delivery, maternal age, HIV status, mode of delivery, birth outcome and birth weight), patients whose maternity charts were not available were comparable to patients included in this analysis. The HIV prevalence in the 236 women who delivered preterm was 48.5% (95%CI 41.9–55.1) and 92.5% (95%CI 85.8–96.7) of the 113 HIV-infected women were on antiretroviral treatment at the time of delivery.

Based on predefined criteria, 47 (19.9%; 95%CI 15.0–25.6) women presented with preterm prelabour rupture of membranes (PPROM) which translates to 2.7% (95%CI 1.9–3.4) of all deliveries.

Women with PPROM did not differ from women without PPROM in age, parity and marital status (Table 1). The proportion of women with PPROM with previous pregnancy complications was higher than in the non-PPROM group (71.9% vs 53.1%) but did not attain statistical significance (*p* = 0.072). The antenatal booking status (89.4% vs 93.6%) and mean gestational age at booking (20.6 vs 18.5 weeks) were not significantly different in either PPROM or non-PPROM groups (Table 1). There was a large difference in proportions of women with a history of smoking between the PPROM and non-PPROM groups but this was not statistically significant. And there were no statistically significant differences in other modifiable risk factors such as BMI and alcohol consumption in the PPROM and non-PPROM groups. Of the 47 PPROM cases, 23 (48.9%) vs 90 (47.6%) of the non-PPROM cases were HIV-infected (*p* = 1.00).

**Table 1:** Characteristics of Women with and without Prelabour Rupture of Membranes at Preterm Delivery

	Prelabour Rupture of Membranes *N* = 47	No Prelabour Rupture of Membranes *N* = 189	Odds Ratio (95%CI)	*p* value
Age (years) Mean (SD)	26.9 (6.6)	26.4 (6.8)		0.606
Category N (%)				
≤ 19	9 (19.2)	32 (16.9)		0.830
> 19	38 (80.8)	157 (83.1)	1.2 (0.5–2.6)	
Parity Mean (SD)	1.3 ± 1.3	1.3 ± 1.3		0.607
Category N (%)				
0	17 (36.2)	61 (32.3)	0.9 (0.5–1.9)	1.000
≥ 1	30 (63.8)	128 (67.7)		
Previous Pregnancy Complications N (%)				
Yes	23 (71.9)	68 (53.1)	2.3 (0.9–5.3)	0.072
No	7 (23.3)	60 (46.9)		
Antenatal Booking N (%)				
Yes	42 (89.4)	175 (93.6)	0.7 (0.2–2.3)	0.745
No	5 (10.6)	12 (6.4)		
Missing Data	–	2		
Gestational Age at Booking (weeks) Mean (SD)	20.6 ± 6.7	18.5 ± 7.4		0.103
Late ANC Booking (≥20wk) N (%)				
Yes	26 (61.9)	85 (48.6)	1.7 (0.9–3.4)	0.126
No	16 (38.1)	90 (51.4)		
Body Mass Index Mean (SD)	29.3 ± 7.8	27.9 ± 5.8		0.562
BMI Category N (%)				
< 30	17 (60.7)	96 (70.6)	1.5 (0.7–3.6)	0.370
≥ 30	11 (39.3)	40 (29.9)		
Missing data	9	53		
History of Smoking N (%)				
Yes	6 (13.6)	11 (6.1)	2.4 (0.9–6.9)	0.111
No	38 (86.4)	169 (93.9)		
Missing data	3	9		
Alcohol Intake N (%)				
Yes	3 (6.8)	16 (8.9)	0.8 (0.2–2.7)	0.773
No	41 (93.2)	164 (91.1)		
Missing data	3	9		
HIV Infection N (%)				
Positive	23 (48.9)	90 (47.6)	1.0 (0.5–1.9)	1.000
Negative	24 (51.1)	96 (50.8)		
Unknown Status		3 (1.6)		
Syphilis N (%)				
Positive	0 (0)	7 (3.8)	–	0.352
Negative	43 (100)	177 (96.2)		
Missing data	4	5		

While there were no stillbirths in the PPROM group, there were 28 (15%) stillbirths in the non-PPROM group (*p* < 0.05) (Table 2). The proportion of infants weighing < 2500 g or born < 34 weeks of gestation was also significantly higher in the non-PPROM group when compared to the PPROM group. The proportion of stillbirths in the non-PPROM group was similar between HIV-infected and HIV-uninfected women (16.9% vs 15.8%; *p* = 1.000) (Table 3). Other adverse birth outcomes in the non-PPROM group such as low birth weight (< 2500 g), very preterm birth (< 34 weeks) and low Apgar scores were more frequent among HIV-uninfected women than HIV-infected women but not attaining statistical difference (Table 3). There were no differences in the proportion of low birth weight (OR 1.0 95%CI 0.3–3.4) or severe preterm birth (OR 3.2; 95%CI 0.9–10.7) between the HIV-infected and HIV-uninfected women whose pregnancies were complicated by PPROM (Table 3).

**Table 2:** Pregnancy Outcomes of Women with and without Prelabour Rupture of Membranes

	Prelabour Rupture of Membranes *n* = 47	No Prelabour Rupture of Membranes *n* = 189	OddsRatio (95%CI)	*p* value
Mode of Delivery N (%)				
Spontaneous Vaginal	25 (53.2)	86 (46.2)	0.7 (0.4–1.3)	0.320
Cesarean	22 (46.8)	100 (53.7)		
Missing data	0	3		
Birth Outcomes N(%)				
Stillbirth	0 (0)	28 (15.0)	–	0.004
Livebirth	47 (100.0)	159 (85.0)		
Birth Weight (g) Mean (SD)	2215 (539)	1944 (676)		0.027
Category N (%)				
< 2500	27 (57.4)	129 (73.3)	0.5 (0.2–0.9)	0.041
≥ 2500	20 (42.56)	46 (26.7)		
Missing data	0	4		
Gestational Age at Delivery-Weeks Median (IQR)	34 (30–38)	33 (29–37)		0.139
Category N (%)				
≤ 34	21 (44.7)	128 (68.1)	0.6 (0.3–1.1)	0.122
> 34	26 (55.3)	60 (31.9)		
Missing data	0	1		
Newborn Apgar Score − 5 mins N (%)				
8–10	41 (91.1)	142 (92.8)	0.8 (0.2–2.6)	0.750
< 8	4 (8.9)	11 (7.2)		
Missing data	2	6

**Table 3:** Pregnancy Outcomes in HIVinfected and Uninfected Women with and without PPROM

	Prelabour Rupture of Membranes		No Prelabour Rupture of Membranes	
HIV Status	Positive (*n* = 23)	Negative (*n* = 24)	Positive (*n* = 90)	Negative (*n* = 96)
Mode of Delivery N (%)				
Spontaneous Vaginal	13 (56.5)	12 (50)	37 (41.6)	48 (51.1)
Cesarean	10 (43.5)	12 (50)	52 (58.4)	46 (48.9)
*Odds Ratio (95%CI)* *p Value*	0.9 (0.3–3.2) 1.000		0.7 (0.4–1.2) 0.236	
Birth Outcomes N (%)				
Still Births	0 (0.0)	0 (0)	15 (16.9)	15 (15.8)
Live Birth	23 (100)	24 (100)	74 (83.2)	80 (84.2)
*Odds Ratio (95%CI)* *p value*	–		0.9 (0.4–2.1) 1.000	
Birth Weight (g)				
Mean (SD)	2180 ± 499	2246 ± 583	2050 ± 674	1842 ± 661
*p value*	0.796		0.043	
Birth Weight by category N (%)				
< 2500	14 (60.9)	13 (54.2)	58 (67.4)	69 (80.2)
≥ 2500	9 (39.1)	11 (45.8)	28 (32.6)	17 (19.8)
*Odds Ratio (95%CI)* *p value*	1.0 (0.3–3.4) 1.000		0.5 (0.3–1.0) 0.082	
Gestational Age at Delivery				
Mean (SD)	32.9 ± 2.3)	33.5 ± 3.8)	33.0 ± 2.9	32.4 ± 2.9
*p value*	0.113		0.089	
Gestational Age by category N (%)				
≤ 34 weeks	13 (56.5)	8 (33.3)	58 (64.4)	68 (70.8)
> 34 weeks	10 (43.5)	16 (66.7)	32 (35.6)	28 (29.2)
*Odds Ratio (95%CI)* *p value*	3.2 (0.9–10.7) 0.080		0.8 (0.4–1.4) 0.433	
Newborn Apgar Score – 5 mins N (%)				
8–10	20 (90.9)	21 (91.3)	70 (95.9)	69 (89.6)
< 8	2 (9.1)	2 (8.7)	3 (4.1)	8 (10.4)
*Odds Ratio (95%CI)* *p value*	0.9 (0.1–7.4)1.000		2.7 (0.7–10.6)0.211	

Eighteen (94.7%) and 81 (92.0%) HIV-infected women in the PPROM and non-PPROM groups respectively received cART (OR 1.6; 95%CI 0.2–13.4) (Table 4). HIV-infected women in the PPROM and non-PPROM groups did not differ significantly by CD4+ count (OR 1.3; 95%CI 0.4–4.3) or HIV-1 viral load (OR 0.5; 95%CI 0.1–1.9). At birth, HIV-1 PCR results were available for 22 of the 23 HIV-exposed babies in the PPROM group and 89 of the 90 babies in the non-PPROM group. None in the PPROM group and only one (1.1%) HIV-exposed baby in the non-PPROM group tested positive at birth.

**Table 4:** HIV-Related Characteristics and Perinatal HIV Infection in association with PPROM

	Prelabour Rupture of Membranes *n* = 23	No Prelabour Rupture of Membranes *n* = 90	Odds Ratio (95%CI)	*p* value
Receiving cART n(%)				
Yes	18 (94.7)	81 (92.0)	1.6 (0.2–13.4)	1.000
No	1 (5.3)	7 (8.0)		
Missing data	4	2		
CD4 Count Median (IQR)	369 (333–656)	508 (325–659)		0.368
CD4 Category N (%)				
< 350	5 (33.3)	18 (28.1)		
≥ 350	10 (66.7)	46 (71.9)	1.3 (0.4–4.3)	0.755
Missing data	8	36		
HIV Viral Load N(%)				
< 1000 copies/ml	7 (63.6)	48 (78.7)		
≥ 1000 copies/ml	4 (36.4)	13 (21.3)	0.5 (0.1–1.9)	0.440
Missing data	12	29		
Neonatal HIV PCR N (%)				
Negative	22 (100)	88 (98.9)	–	
Positive	0	1		
Missing data	1	1		

## Discussion

In this most recent study in a high HIV endemic setting in South Africa, the preterm birth rate is approximately 17% and one in five of these preterm births is further complicated by prelabour rupture of membranes. HIV infection itself was not associated with increased risk of PPROM, and adverse birth outcomes. Gestational age at delivery and birth weight were not different in HIV-infected women compared to HIV-uninfected women whose pregnancies were complicated by PPROM. In addition, there was no evidence of increased mother-to-child transmission of HIV in women receiving cART and whose pregnancies were complicated by PPROM.

In current times when cART is the standard of care, we report a preterm delivery rate of 16.8%, almost 50% were HIV-infected and 90% were on cART and virally suppressed. In a similar maternity audit at another local regional hospital in 2014, 32.2% of all deliveries were classified as preterm (< 37 weeks) [19]. At the time of the latter audit, only 45% of women were on cART prior to delivery. A reduction in preterm deliveries was reported for a rural setting in the region from 16.3% in 2010 to 9.3% in 2015, and the reduction was attributed to the rollout of the cART program for pregnant women in the region [20]. The higher preterm delivery rate in our study is more likely due to the facility serving as a referral centre for high risk pregnancies in this urban region.

Almost one in five (19.9%) of the preterm deliveries at this regional facility was further complicated by prelabour rupture of membranes, which translates to 2.67% (95%CI 1.92–3.42) of all deliveries. Our incidence of PPROM (2.67%) in 2018 in a high HIV endemic region in South Africa is similar to findings reported for the US (2.4%) or France (2–3%) where the antenatal HIV prevalence is markedly lower [18, 21]. In the only other study conducted in KwaZulu Natal in 2013, prior to the cART rollout for pregnant women, 130 (1.69%; 95%CI 1.4–1.98) of 7694 deliveries were complicated by PPROM [22]. Although intra-amniotic, urinary tract and sexually transmitted infections have long been identified as a cause of PPROM [23], our retrospective analysis did not allow for further exploration of the role of other infections in PPROM. We can however speculate the possible role of STIs in our study because this population is known for its high prevalence of STIs (32.3%), mostly asymptomatic and could have likely contributed to the PPROM incidence in this population [24]. The STI study in the same population demonstrated the strong likelihood of an association between asymptomatic and untreated STIs and preterm births [24]. Another study implicated the abnormal vaginal microbiome that is less dominated by Lactobaccilli sp. as a risk for PPROM [25]. Here again, BV testing is not routinely performed in our setting, but the high BV prevalence recently reported for this population needs further investigation for its role in preterm births and PPROM [26].

Our hypothesis that PPROM incidence is higher among HIV-infected women was not supported by our findings of similar high HIV positivity rates between women in the PPROM group and the non-PPROM group. Furthermore, the proportion of women on cART or with lower CD4 count or higher viral load was equally distributed among women with PPROM or without PPROM. The overall high HIV seroprevalence in our cohort is consistent with that reported in another PPROM study in the KZN region and the most recent national antenatal HIV survey [22, 27]. Unfortunately, the other KZN study lacked a control group of preterm deliveries without PPROM. From our study alone, it does not appear that HIV- infected women receiving cART were at a higher risk for preterm prelabour rupture of membranes.

Besides infection, other modifiable factors such as BMI and smoking have also been implicated as risk factors for PPROM. In our study, 22% of the women with preterm deliveries were obese but obesity was not characteristically unique to women with PPROM or non-PPROM. Our findings are supported by other studies that have confirmed that high BMI did not modify the risk for PPROM [28, 29]. The study by Dekker et al. in New Zealand, however reported that a low BMI (< 20) nearly doubled the risk for PTB complicated by PPROM in nulliparous women [29]. While tobacco smoking is a known risk factor for PTB, smoking in our population is less common when compared to non-African populations, and although the proportion of women with a history of smoking was two-fold higher in the PPROM group than the non-PPROM group, this observation was not statistically significant [30].

In our study, women with PPROM were two times as likely to have had complications in a previous pregnancy than women without PPROM. The most common complications included preterm births (18.8%), abruptio placentae (15.6%) and preeclampsia (15.6%) and these complications were more common among women with PPROM but not significantly different from the non-PPROM group. In a recent Thailand study, women with a history of preterm birth in a previous pregnancy were three times at higher risk of presenting with PPROM, while multiparity appeared to be protective against PPROM [31]. Our study sample size was limited, and we were unable to support or refute the Thailand findings.

Overall, birth outcomes in our study population were better in the PPROM group than the non-PPROM group. There were no stillbirths in the PPROM group and a significantly higher proportion of stillbirths among women without PPROM which could have been due to other obstetric complications such as maternal hypertensive disorders, antepartum haemorrhage or fetal invasive bacterial infection [32]. Similarly, despite no difference in the gestational age between both groups, the PPROM group had a significantly lower proportion of newborns weighing < 2500 g. There was no evidence in our study of HIV contributing to worse pregnancy outcomes in the PPROM group. Although HIV-infected women with PPROM were two times as likely to deliver < 34 weeks when compared to their HIV-uninfected counterparts, this trend was not significant. There was a lower cesarean delivery rate among women with PPROM when compared to the non-PPROM group, and comparable between HIV-infected and uninfected women. Inconsistent with our high cesarean rate, the other KZN PPROM study reported a higher vaginal delivery rate among women with PPROM but did not compare this with other preterm deliveries [22]. Nonetheless, similar to our findings, there was no evidence of worse pregnancy and newborn outcomes among HIV-infected women with PPROM when compared to their HIV-uninfected counterparts [22]. As in our study, most HIV-infected women in the study by Msomi et al. were also on cART prior to delivery [22]. The other positive effect of cART in HIV-infected women in our cohort is evidenced by the zero mother-to-child transmission rate of HIV in the PPROM group, and only one case of in-utero transmission in the non-PPROM group. The neonate tested positive at birth, an indication of an in-utero infection. Our findings are supported by another study that reported women with PPROM on cART or HAART are less likely to transmit HIV infection to the infant [17]. This is in contrast to a US study conducted in the early 90’s (pre-ARV era) that reported a four-fold increased risk of MTCT in women who developed PPROM [33].

## Study limitations

Our sole reliance on data extracted from maternity records resulted in missing data for certain study participants but this was distributed equally among both PPROM and non-PPROM groups. The small proportion of PPROM cases was an additional limitation. A further limitation to our study was that we did not extend our observation to beyond the immediate postpartum period and hence neglected to include neonatal outcomes including perinatal HIV diagnosis after birth. We conducted this study at a regional hospital which also served as a referral centre for high risk pregnancies in the local area, hence our findings may not be generalizable to the larger population of the KZN.

## Conclusion

In this most recent study of preterm deliveries in a high HIV endemic setting, there was no evidence of an association of PPROM with HIV infection, and neither were HIV-infected women who were receiving cART and who presented with PPROM at a higher risk for adverse pregnancy outcomes and MTCT. Based on our findings, it seems that standard management protocols for PPROM could remain unchanged for HIV-infected women on cART and virally suppressed.

## Data Availability

Dataset is available upon request.

## Abbreviations

HIV: Human immunodeficiency virus
cART: combination antiretroviral treatment
PTB: Preterm birth
PPROM: Preterm prelabour rupture of membranes
KZN: KwaZulu Natal
HAART: Highly active antiretroviral treatment
MTCT: Mother-to-child transmission
BMI: Body mass index
PCR: Polymerase Chain Recation
